# The complete chloroplast genome of *Cymbidium eburneum* (Orchidaceae)

**DOI:** 10.1080/23802359.2019.1629844

**Published:** 2019-07-16

**Authors:** Meng Wang, Jian-Bing Chen, Gui-Zhen Chen, Li- Jun Chen, Xin-Yi Wu, Jie Huang

**Affiliations:** a Key Laboratory of National Forestry and Grassland Administration for Orchid Conservation and Utilization, Shenzhen, China;; b Shenzhen Key Laboratory for Orchid Conservation and Utilization, The National Orchid Conservation Centre of China and The Orchid Conservation and Research Centre of Shenzhen, Shenzhen, China

**Keywords:** *Cymbidium eburneum*, chloroplast genome, phylogenetic, endangered species, Orchidaceae

## Abstract

*Cymbidium eburneum* Lindl. is an endangered species of Orchidaceae and distributed in Guangxi, Yunnan and Hainan of China, and India, Myanmar, Nepal, Vietnam. Here, we report the complete chloroplast (cp) genome sequence and the cp genome features of *C. eburneum.* The complete chloroplast (cp) genome sequence of *C. eburneum* is 156,520 bp in length and presented a typical quadripartite structure including one large single-copy region (LSC, 85,518 bp), one small single-copy region (SSC, 20,014 bp), and two inverted repeat regions (IRs, 25,471 bp). The cp genome encoded 135 genes, of which 106 were unique genes (78 protein-coding genes, 24 tRNAs, and 4 rRNAs). The phylogenetic relationships show that *C. eburneum* is closely related to other species in the genus *Cymbidium*, and is sister with *C. tracyanum*.

Since the genus *Cymbidium* was established by Swartz (1799), some infrageneric treatments have been published (Berg 2002; Liu et al. 2006; Chen et al. 2009, 2019; Zhang et al. 2019). At present, approximately 80 species are recognized within the genus. This genus is mainly distributed in tropical, subtropical Asia, Papua New Guinea and Australia (Chen et al. 2009, Pridgeon et al. 2009). In China, more than 50 species are found and 19 of them are endemic (Liu et al. 2006; Chen et al. 2009). *Cymbidium* is one of the best known and most widely grown of orchid in worldwide horticulture.

The genomic studies may contribute to the study of species identification, germplasm diversity, and genetic engineering (Lin et al. 2015). The complete chloroplast genome sequence of *C. eburneum* was assembled in this study.

Leaf samples of *C. eburneum* were obtained from the Orchid Conservation and Research Centre of Shenzhen, and specimens were deposited in the National Orchid Conservation Center herbarium (NOCC; specimen code Z.J.Liu 2625). Total genomic DNA was extracted from fresh material using the modified CTAB procedure of Doyle and Doyle (1987). Sequenced on Illumina Hiseq 2500 platform (San Diego, CA). Genome sequences were screened out and assembled with MITObim v1.8 (Hahn et al. 2013), which resulted in a complete circular sequence of 156,520 bp in length. The cp-genome was annotated with CpGAVAS (Liu et al. 2012).

The cp genome sequence of *C. eburneum* (GenBank accession MK820374) is 156,520 bp length and presented a typical quadripartite structure including one large single-copy region (LSC, 85,518** **bp), one small single-copy region (SSC, 20,014 bp), and two inverted repeat regions (IRs, 25,471 bp). The cp genome encoded 135 genes, of which 106 were unique genes (79 protein-coding genes, 23 tRNAs, and 4 rRNAs).

To determine the phylogenetic position of *C. eburneum*, we used 12 accessions of *Cymbidium* for molecular analysis, two species from related genera were used as outgroups. The phylogenetic tree was constructed based on the maximum-likelihood (ML) methods. The ML analysis was performed using the CIPRES Science Gateway web server (RAxML-HPC2 on XSEDE 8.2.10) with 1000 bootstrap replicates and settings as described by Stamatakis et al. (2008). The result showed that they were all clustered together, and *C. eburneum* is mostly related to taxa with *C. tracyanum* (Figure 1). This newly reported chloroplast genome will be helpful for further study on phylogenetic study, species identification, and genetic engineering.

**Figure 1.**
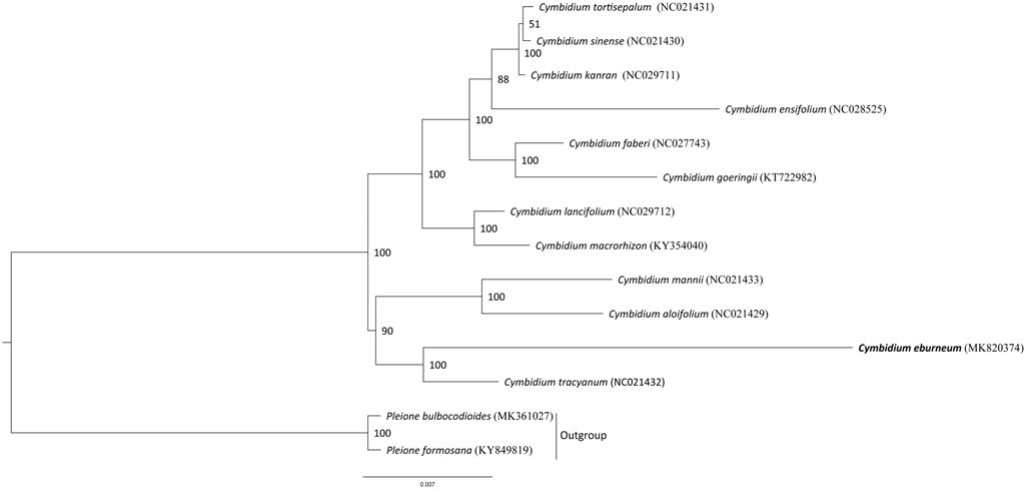
Phylogenetic position of *C. eburneum* inferred by maximum likelihood (ML) of complete cp genome. The bootstrap values are shown next to the nodes.
